# Low-dose rapamycin does not impair vascular integrity and tubular regeneration after kidney transplantation in rats

**DOI:** 10.1038/s41598-021-95790-1

**Published:** 2021-08-11

**Authors:** Uwe Hoff, Denise Markmann, Melina Nieminen-Kelhä, Klemens Budde, Björn Hegner

**Affiliations:** 1 Department of Nephrology and Critical Care Medicine, Charité – Universitätsmedizin Berlin, Corporate Member of Freie Universität Berlin, Humboldt-Universität zu Berlin, and Berlin Institute of Health, Berlin, Germany; 2 Nieren- und Dialysezentrum Schöneberg-Tempelhof, Berlin, Germany; 3 Department of Neurosurgery, Charité – Universitätsmedizin Berlin, Corporate Member of Freie Universität Berlin, Humboldt-Universität zu Berlin, and Berlin Institute of Health, Berlin, Germany; 4 Vitanas Hospital for Geriatric Medicine, Berlin, Germany

**Keywords:** Nephrology, Acute kidney injury

## Abstract

mTOR inhibitors offer advantages after kidney transplantation including antiviral and antitumor activity besides facilitating low calcineurin inhibitor exposure to reduce nephrotoxicity. Concerns about adverse effects due to antiproliferative and antiangiogenic properties have limited their clinical use particularly early after transplantation. Interference with vascular endothelial growth factor (VEGF)-A, important for physiologic functioning of renal endothelial cells and tubular epithelium, has been implicated in detrimental renal effects of mTOR inhibitors. Low doses of Rapamycin (loading dose 3 mg/kg bodyweight, daily doses 1.5 mg/kg bodyweight) were administered in an allogenic rat kidney transplantation model resulting in a mean through concentration of 4.30 ng/mL. Glomerular and peritubular capillaries, tubular cell proliferation, or functional recovery from preservation/reperfusion injury were not compromised in comparison to vehicle treated animals. VEGF-A, VEGF receptor 2, and the co-receptor Neuropilin-1 were upregulated by Rapamycin within 7 days. Rat proximal tubular cells (RPTC) responded in vitro to hypoxia with increased VEGF-A and VEGF-R1 expression that was not suppressed by Rapamycin at therapeutic concentrations. Rapamycin did not impair proliferation of RPTC under hypoxic conditions. Low-dose Rapamycin early posttransplant does not negatively influence the VEGF network crucial for recovery from preservation/reperfusion injury. Enhancement of VEGF signaling peritransplant holds potential to further improve outcomes.

## Introduction

Although rapamycin (Rapa), the first-in-class inhibitor of the mechanistic target of rapamycin (mTOR), had been approved for immunosuppression after kidney transplantation more than 20 years ago the debate on the most appropriate use of mTOR inhibitors (mTORi) is still ongoing^1,2^. During the last decades it became clear that beyond immunosuppression mTORi exert not only specific anti-viral activity resulting in reduced incidents of cytomegalovirus^3–5^ and BK virus^4^ infections but also provide some defense against malignant tumors^5^. Moreover, it had been speculated that mTORi could protect from cardiovascular events and chronic allograft dysfunction through intrinsic mechanisms and by enabling avoidance or at least reduction of nephrotoxic calcineurin inhibitors (CNI) with the final goal of improved long-term graft and patient survival.

Despite these important advantages, mTORi are frequently withheld or withdrawn since they are perceived as being associated with an unfavorable safety profile limiting their use in many transplant recipients. In particular, delayed wound healing, formation of lymphoceles, increased proteinuria, higher rejection rates, cytopenias, an adverse metabolic profile and increased mortality have been of concern^6,7^. Studies yielded heterogeneous results depending on timing of mTORi administration (de novo transplants, early conversion, late conversion) and choice of concomitant immunosuppression (mycophenolate, CNI at standard or recued doses, steroids). Of note, recent trials and meta-analyses clearly demonstrate non-inferiority of mTORi based regimes that acknowledge drug-drug interactions and combine mTORi and CNI at reduced doses^8–10^ indicating a learning curve that may not have reached its climax yet.

mTOR, by integrating nutrient availability and growth factor signaling, functions as a ubiquitous central regulator of proliferation, protein synthesis and other important cell functions such as autophagy^11^. Many of the advantageous but also of the adverse non-immune effects of mTORi have been attributed to their inherent antiproliferative properties and their complex context dependent impact on cellular growth, differentiation, and metabolism^11^. One example is the antitumor effect of mTORi that has been ascribed not only to general inhibition of proliferation but also to a specific antiangiogenic activity due to reduced production of and response to vascular endothelial growth factor (VEGF)-A resulting in limited blood supply to the tumor^12,13^.

Importantly, expression of VEGF-A and its receptors is also found with distinctive patterns at multiple sites in healthy kidneys such as endothelial cells of glomeruli and peritubular capillaries, podocytes, mesangial cells and tubular epithelial cells^14^. The VEGF network is involved in maintaining physiologic functioning of glomeruli and peritubular capillaries^14,15^ and has been found to be dysregulated in a large variety of kidney diseases including diabetic nephropathy, glomerulonephritis, thrombotic microangiopathies and chronic allograft nephropathy (CAN) after kidney transplantation^14,15^. Remarkably, also tubular epithelial cells feature a functional VEGF network^14,16^ connecting them to vascular maintenance under stable conditions and initiation of angiogenesis when oxygen supply does not meet the demand^15^. Moreover, VEGF-A has been implicated in proliferation and protection of tubular cells from apoptosis rendering it a survival factor for tubular epithelium^16^ that might act in an autocrine or paracrine manner.

To further improve mTORi containing immunosuppressive regimes for de novo kidney transplants, we aimed to study the impact of mTOR inhibition with Rapa on vascular integrity and tubular regeneration in correlation to the VEGF network during the immediate posttransplant period in a life-supporting rat kidney transplantation model. We found intact vascular structures, adequate tubular cell proliferation, and rapid functional recovery from preservation/reperfusion injury until day 2 in the face of Rapa treatment. VEGF-A, VEGF receptor 2 (VEGF-R2) and the VEGF co-receptor Neuropilin-1 were upregulated by Rapa within 7 days. In addition, rat proximal tubular cells (RPTC) responded to hypoxia in vitro with increased VEGF-A and VEGF-R1 expression that was not suppressed by Rapa at therapeutic concentrations. These findings support the introduction of mTORi immediately after transplantation and provide insight into the early dynamics of the VEGF network opening a perspective for targeted interventions to further improve outcomes.

## Results

We took advantage of our rat kidney transplantation model with a low-responder strain combination (Fischer to Lewis)^20^ to study the effect of mTOR inhibition with Rapa on vascular integrity as well as tubular and functional recovery during the immediate posttranplantation phase.

### Rapa does not interfere with vascular integrity during recovery from posttransplantation preservation injury

**Figure 1:**
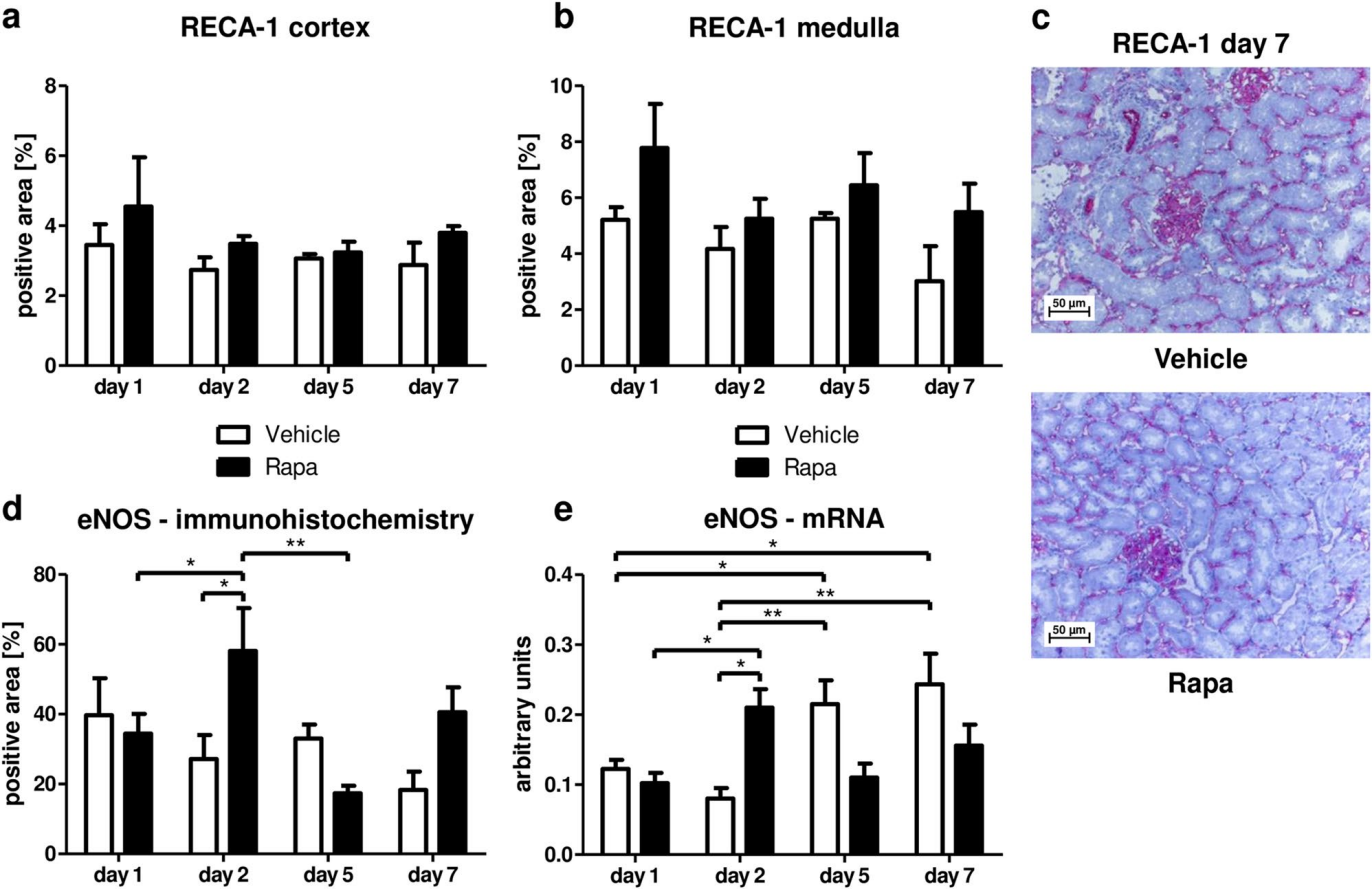
Effect of Rapa on vascular integrity early after kidney transplantation. (**a**, **b**) Positive area in the cortex (**a**) and medulla (**b**) of transplanted kidneys from vehicle or Rapa treated rats stained by immunohistochemistry for rat endothelial cell antigen (RECA)-1. n = 4. (**c**) Representative photomicrographs for RECA-1 immunohistochemistry 7 days posttransplant with vehicle or Rapa treatment. (**d**) Total positive area in kidney transplants from vehicle or Rapa treated rats stained by immunohistochemistry for endothelial nitric oxide synthase (eNOS). n = 4–5. (**e**) Quantification of eNOS mRNA in whole transplanted kidneys by real-time PCR after treatment with vehicle or Rapa. n = 4–5. *P < 0.05, **P < 0.01.

As shown by immunostaining of endothelial cells, mTOR inhibition did not compromise the integrity of glomerular or peritubular capillaries neither in cortex (Fig. 1a, c) nor in outer medulla (Fig. 1b) that is particularly susceptible to hypoxic injury. Furthermore, expression of eNOS was even increased on day 2 in Rapa treated animals compared to vehicle on protein (Fig. 1d) and mRNA levels (Fig. 1e) indicating endothelial protection in the post-immediate period. On days 5 and 7, representing the late regeneration phase, eNOS mRNA but not protein was upregulated in vehicle treated animals in comparison to the early phase. However, no significant difference was detected between both treatment groups (Fig. 1d, e).

**Figure 2:**
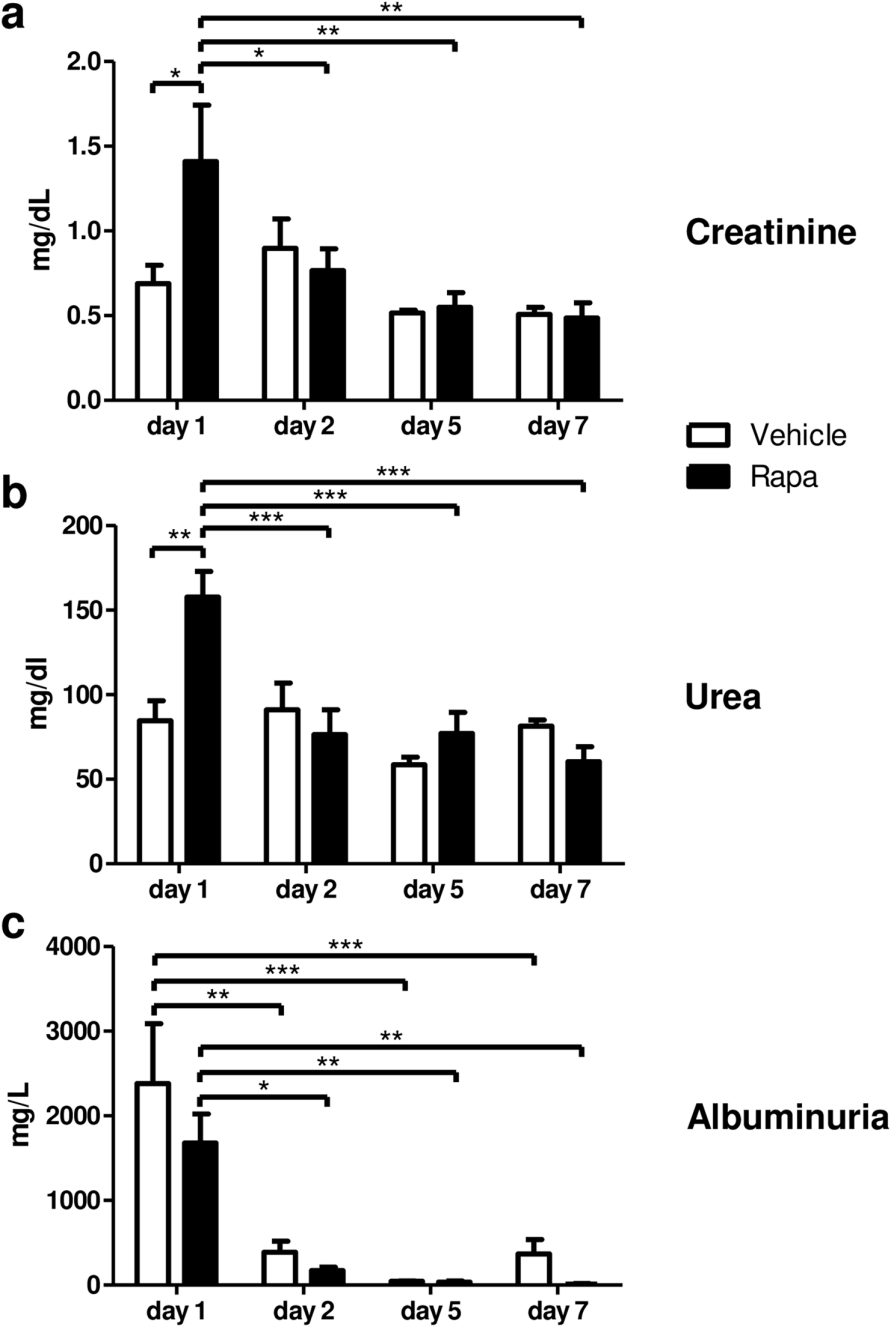
Renal function and albuminuria during the first week after kidney transplantation. Plasma creatinine (**a**) and urea (**b**) concentrations in transplanted rats receiving vehicle or Rapa. n = 5–7. (**c**) Albumin concentrations measured in urine samples collected over 24 h in metabolic cages. n = 4–5. *P < 0.05, **P < 0.01, ***P < 0.001.

On a physiologic level, kidney function was mildly impaired in Rapa treated animals on day 1 but completely recovered as early as on day 2 as reflected by plasma creatinine (Fig. 2a) and urea measurements (Fig. 2b). There was moderate albuminuria on day 1 that completely resolved until day 5 independently of treatment (Fig. 2c). Control rats merely developed slight albuminuria on day 7 not significantly different from rats on Rapa (Fig. 2c). These findings suggest rapid resolution of functional changes induced by preservation/reperfusion injury in recipient rats despite mTOR blockade with Rapa.

### The intrarenal VEGF network is activated by Rapa early posttransplant

**Figure 3:**
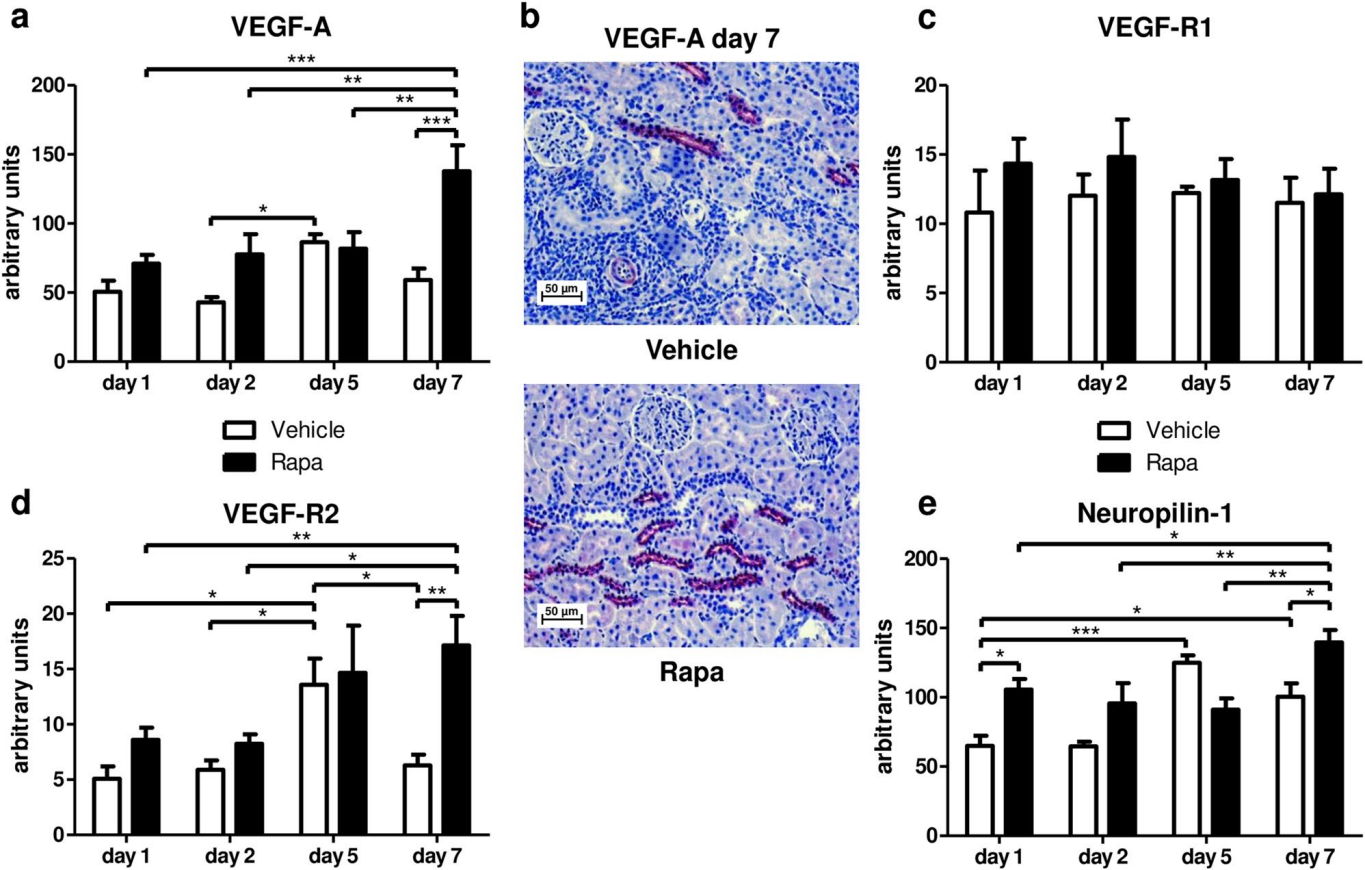
The vascular endothelial growth factor (VEGF) network in kidney grafts from rats receiving vehicle or Rapa. Quantitative real-time PCR on days 1–7 (**a**) and immunohistochemistry on day 7 (**b**) for VEGF-A. Representative photomicrographs are shown. mRNA expression of the VEGF receptors 1 (VEGF-R1; **c**) and 2 (VEGF-R2; **d**) and the VEGF co-receptor neuropilin-1 (**e**) as assessed with quantitative real-time PCR. n = 4–6. *P < 0.05, **P < 0.01, ***P < 0.001.

Hypoxia inducible factor-1α (HIF-1α) is stabilized under hypoxic conditions as found during transplantation associated ischemia and has been shown to be activated by mTOR^21^. As a transcription factor, HIF-1α promotes expression of VEGF-A, the decisive mediator of vascular homeostasis and driver of angiogenesis. Thus, we analyzed the VEGF network to decipher autocrine and paracrine cues with the ability to maintain peritransplant vascular integrity found in our model. VEGF-A mRNA transcripts were 2.3 times more abundant in Rapa treated rats compared to vehicle controls on day 7 and were already increased by trend on days 1 and 2 (Fig. 3a). Remarkably, VEGF-A transcripts in vehicle treated rats increased significantly from day 2 to day 5 to equal those of Rapa treated rats only on day 5 (Fig. 3a). VEGF expression was predominantly found in epithelial cells of distal tubules as demonstrated by immunohistology (Fig. 3b). VEGF-R1 mRNA was neither altered by treatment nor time (Fig. 3c). Of note, expression of VEGF-R2 paralleled that of VEGF-A (Fig. 3d): Rapa was associated with nonsignificant higher levels on days 1 and 2 and 2.7-fold more transcripts on day 7 in comparison to vehicle. On day 5, there were significantly more VEGF-R2 transcripts in vehicle treated animals than at the other time points reaching the same level as found with Rapa. A similar pattern was observed for neuropilin-1, a co-receptor for VEGF-A (Fig. 3e). Rapa in comparison to vehicle resulted in significantly increased transcription of neuropilin-1 on days 1 and 7 and by trend on day 2 while expression of neuropilin-1 was upregulated on day 5 in the vehicle group (Fig. 3e).

### Stimulation of VEGF expression by hypoxia overrides inhibitory Rapa mediated effects at therapeutic concentrations

**Figure 4:**
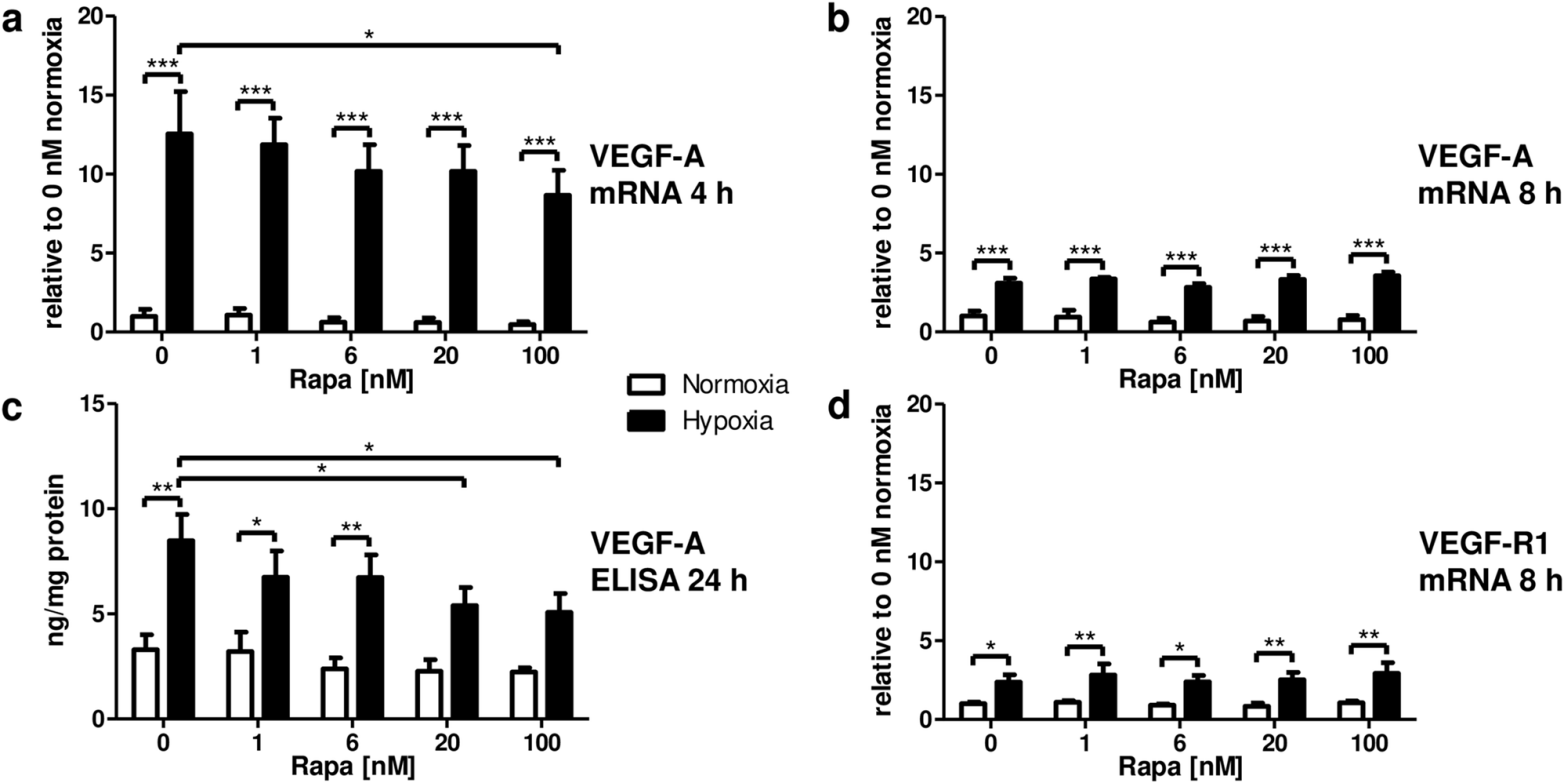
Influence of hypoxia and mTOR inhibition by Rapa on the vascular endothelial growth factor (VEGF) network in rat renal proximal tubular cells (RPTC). RPTC were incubated with Rapa under normoxic or hypoxic conditions. Expression of VEGF-A mRNA was analyzed by quantitative real-time PCR after 4 h (**a**) and after 8 h (**b**). Results were normalized to vehicle control (0 nM Rapa) at normoxia (set to 1.00). Normoxia n = 7–8, hypoxia n = 4. (**c**) VEGF-A protein measured with an enzyme-linked immunosorbent assay (ELISA) in cell culture supernatants after 24 h normalized to total protein content. n = 9. (**d**) Expression of VEGF receptor 1 (VEGF-R1) mRNA quantified by real-time PCR after 8 h normalized to vehicle control (0 nM Rapa) at normoxia (set to 1.00). Normoxia n = 6, hypoxia n = 5. *P < 0.05, **P < 0.01, ***P < 0.001.

To analyze the relative contribution of hypoxia and mTOR inhibition – both operative in our transplant model—on the VEGF network, proliferation, and metabolism in renal tubular epithelial cells, we performed a series of in vitro experiments with RPTC. VEGF-A mRNA was strongly upregulated 4 h after induction of hypoxia (Fig. 4a). Rapa slightly reduced VEGF-A only at the suprapharmacological dose of 100 nM (Fig. 4a). Relative expression waned after 8 h but was still highly significantly amplified under hypoxic conditions compared to normoxia (Fig. 4b). Elevated mRNA levels translated into increased secretion of VEGF-A into the supernatant as determined after 24 h by ELISA (Fig. 4c). Again, the amount of VEGF-A protein was reduced only at 20 nM and 100 nM (Fig. 4c), concentrations exceeding those found in patient plasma. There was no detectable effect of mTOR inhibition by Rapa on VEGF-A expression under normoxic conditions both on mRNA and protein levels (Fig. 4a-c). VEGF-R1 mRNA was markedly upregulated by hypoxia (Fig. 4d). Rapa did not influence mRNA levels independently of oxygen saturation (Fig. 4d). In accordance with reports on animal studies^14^, we did not detect VEGF-R2 mRNA transcripts in RPTC (not shown).

### Proliferation of tubular epithelial cells is preserved despite mTOR inhibition by Rapa during posttransplant repair and under hypoxia

**Figure 5:**
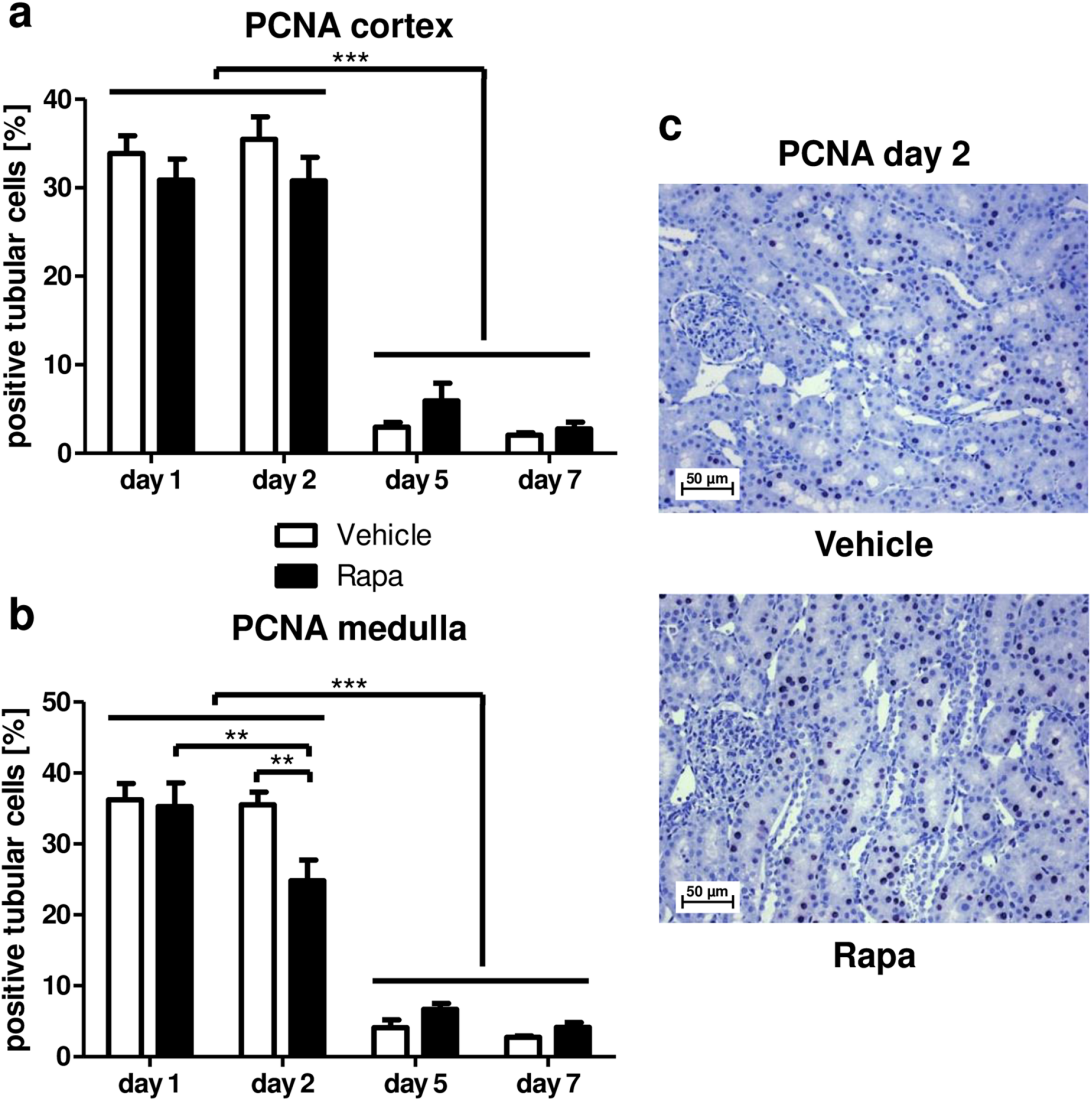
Effect of Rapa on proliferation of tubular cells after kidney transplantation. Percentage of tubular cells positive for proliferating cell nuclear antigen (PCNA) in cortex (**a**) and medulla (**b**) during the first week after kidney transplantation as seen with immunohistochemistry. n = 3. (**c**) Representative photomicrographs for PCNA immunohistochemistry 2 days posttransplant with vehicle or Rapa treatment.

**Figure 6:**
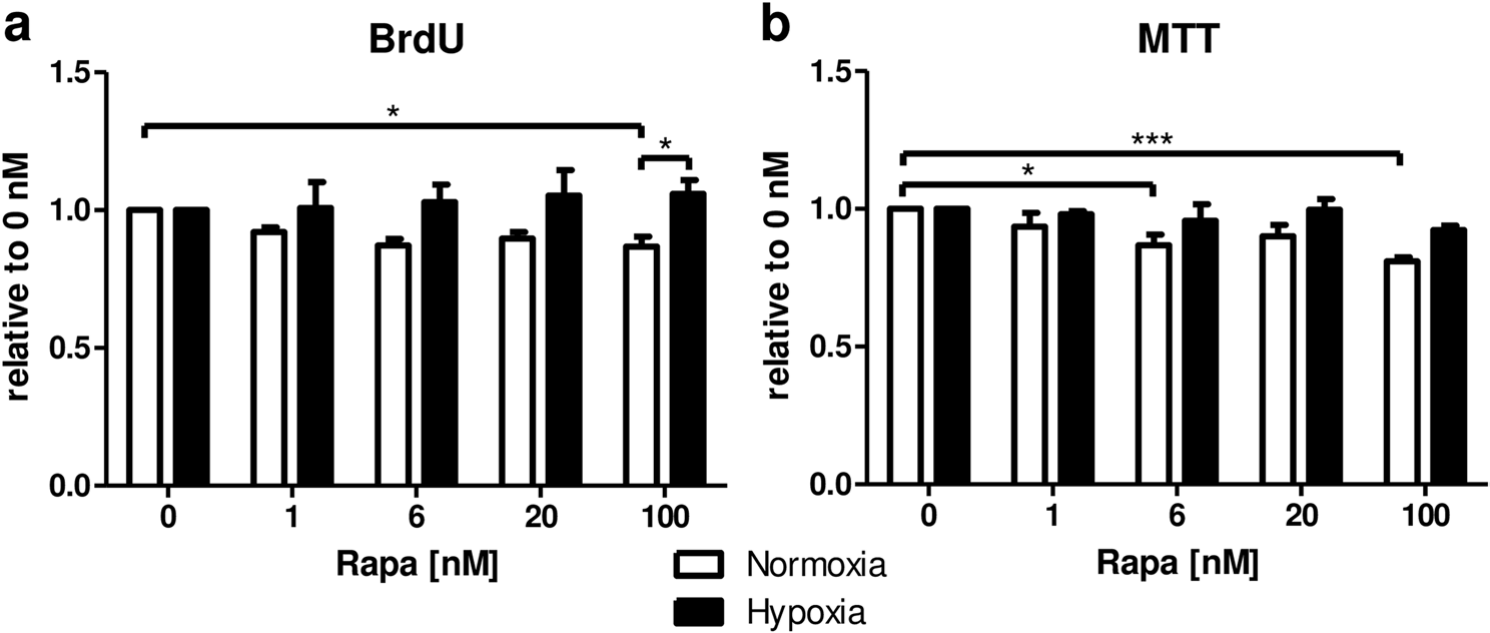
Effect of Rapa on proliferation and metabolic activity of rat renal tubular cells in vitro under normoxic and hypoxic conditions. Rat renal proximal tubular cells (RPTC) were incubated with Rapa under normoxic or hypoxic conditions for 24 h. (**a**) Proliferation was assessed as incorporation of BrdU. Results are normalized to vehicle control (0 nM Rapa) for each experiment. Normoxia n = 6, hypoxia n = 4. (**b**) Metabolic activity was measured as conversion of MTT to formazan. Results are normalized to vehicle control (0 nM Rapa) for each experiment. Normoxia n = 4, hypoxia n = 3. *P < 0.05, **P < 0.01, ***P < 0.001.

More than 30% of tubular cells in cortex and medulla exhibited proliferative activity as determined by immunostaining for proliferating cell nuclear antigen (PCNA) on day 1 after transplantation independently of mTOR inhibition (Fig. 5a–c). On day 2, proliferation remained high in the cortex in both groups (Fig. 5a, b). However, Rapa diminished proliferation of tubular epithelial cells located in the medulla (Fig. 5b). At later time points, proliferation was largely decreased in cortex and medulla in both treatment groups (Fig. 5a–c). Cell culture experiments revealed that proliferation of RPTC as measured by BrdU incorporation was decreased by Rapa only under normoxic conditions whereas mTOR inhibition had no effect when cells were exposed to hypoxia (Fig. 6a). Similarly, overall metabolic activity of RPTC when determined with the MTT assay declined with increasing doses of Rapa at normoxia, but not hypoxia (Fig. 6b).

## Discussion

In our low-responder life-supporting rat renal transplantation model, the mTORi Rapa did not exert adverse effects in the early posttransplant period up to day 7. This is in contrast to early reports that linked induction immunosuppression with Rapa to increased rates of delayed graft function (DGF)^22^ and prolonged recovery from this important complication^23,24^. Since DGF is associated with unfavorable short- and long-term outcomes^25^, it was concluded that Rapa might not be the best choice for transplants at risk for DGF^23^ although 1-year graft function was not compromised^24^. More recent studies did not find an association of mTORi use with DGF^5,26^. The most important differences to earlier protocols are reduced target trough levels for mTORi, e.g. for Rapa instead of 10–15 ng/mL^23^ only 4–8 ng/mL as established in the ELITE-Symphony study^27^, no combination with mycophenolate as an additional anti-proliferative agent^9^, and reduced CNI exposure^28^. Hence, we aimed to elucidate the effect of low-dose Rapa on regenerative mechanisms operative early after transplantation that might be involved in protection from DGF.

A major objection put forward against the use of mTORi in the immediate posttransplant period is a possible negative impact of their well-known antiproliferative potential on graft regeneration from preservation injury resulting in DGF. However, we did not find reduced proliferation with Rapa in tubular epithelial cells in cortex and medulla with the exception of a 30% reduction on day 2 in the medulla at low Rapa concentrations. In addition, inhibition of the VEGF axis by Rapa^12,13^ might be particularly harmful for transplanted kidneys with preservation/reperfusion damage since VEGF-A is not only required for maintaining integrity and functionality of glomerular structures^14,29^ but has also been established as a growth supporter for tubular epithelium^16^, the renal compartment with the highest susceptibility to hypoxic damage. Rapa and other mTORi have been studied in various models relevant for kidney transplantation. However, to our knowledge, none of the previously published works examined the effect of mTOR inhibition on allograft regeneration and the VEGF network in the early posttransplant period.

As an example, Ko et al. used the same low-responder Fischer-to-Lewis kidney transplantation model as us to study the impact of Rapa on chronic allograft dysfunction^30^. After 24 weeks, they did not observe reduced expression of VEGF-A and VEGF-R1 in whole kidney grafts with Rapa in comparison to Cyclosporine A (CsA) while VEGF-R2 mRNA was almost doubled in CsA treated rats^30^. Tubuli were not evaluated separately, but mRNA and protein levels of the VEGF axis components were strikingly reduced in glomerular structures and intrarenal arteries with Rapa treatment^30^. Glomerular and vascular impairment of the VEGF system was associated with increased proteinuria on one hand and protection from vasculopathy on the other^30^, illustrating both sides of the coin with potential detrimental and beneficial consequences of mTORi use after transplantation.

In our study, glomerular and peritubular capillaries were not structurally altered with intact eNOS expression indicating endothelial functioning during the first week after transplantation. Correspondingly, albuminuria resolved as quickly in Rapa treated animals as in those receiving vehicle. Thus, there was no evidence for adverse effects of mTOR inhibition on restoration of glomerular function and vascular integrity immediately after transplantation. Moreover, our in vitro experiments with RPTC demonstrate neutralization of any inhibiting effect of Rapa at clinically relevant concentrations on proliferation and metabolism when cells were exposed to hypoxic conditions. Remarkably, these findings on structural and functional levels were related to preservation or even amplification of the VEGF network in vivo and in vitro despite the presence of Rapa. A possible explanation is the activation of some defense mechanism during cold ischemia overriding potentially negative influences of mTOR inhibition on VEGF production and function as described in other contexts^12,13,30^.

The hypoxia inducible factors (HIF) 1 and 2 have been identified as central players in the protection of tubular epithelial cells during ischemia–reperfusion injury (IRI)^31^. As oxygen-sensitive transcription factors, they promote the expression of proteins related to the adoptive response to hypoxia such as erythropoietin (EPO) and VEGF-A^21^. In rat kidneys, activation of the cellular master switch for adaptation to low oxygen tension, HIF-1α, has been observed not only during ischemia but also on days 3, 5 and 7 of reperfusion without evidence of persistent tissue hypoxia^32^. The HIF-1α target genes EPO and VEGF-A were upregulated 3 days after ischemia and negative genetic interference with HIF-1α directed siRNA exacerbated tubular injury and dramatically worsened renal function on day 3^32^. Thus, induction of HIF-1α and its target genes including VEGF-A appears to be necessary in the immediate and early posttransplant period to ensure timely recovery from preservation/reperfusion injury. Besides hypoxia, oxygen-independent pathways such as growth factor signaling, heat shock protein 90, and others can activate HIF-1α dependent transcription^21^. In proximal tubular cells, such pathways have been shown to be induced independently of oxygen levels by nutrient depletion and replenishment mimicking transplantation associated ischemia^32^. Together with our findings, this evidence suggests that hypoxia in addition to hypoxia independent mechanisms operative in preservation/reperfusion injury act as powerful inducers of the VEGF network that is not suppressed by Rapa at low concentrations.

Following evidence from animal studies, HIF-1α targets have been evaluated as therapeutic options to reduce DGF. Disappointingly, EPO failed to improve recovery from preservation/reperfusion injury and to protect from DGF in transplant patients^33,34^. Different means of HIF-1α induction other than hypoxia such as peritransplant recipient treatment with carbon monoxide^35^ or donor pretreatment with a prolyl-hydroxylase inhibitor^36^ are still experimental. Amplification of VEGF signaling during the vulnerable peritransplant period could be an alternative approach. Application of highly specific VEGF-R2 activating aptamers^37^ directly to the allograft during cold storage or immediately prior to graft implantation would be a reasonable approach. This strategy holds promise to be particularly beneficial since it would specifically target intrarenal vascular, glomerular, and tubular structures at risk for preservation/reperfusion injury with good responsiveness to VEGF within a short window of opportunity while avoiding off-target effects in other tissues.

In human kidney transplant recipients, introduction of mTORi bears a risk for detrimental consequences predominantly in already structurally altered grafts with low glomerular filtration rate and preexisting proteinuria as a consequence of severely damaged glomerula in CAN^38^. Thus, early use of mTORi might be prudent to avoid the development of calcineurin inhibitor toxicity and CAN and to derive the maximum benefit with regard to viral infections, cardiovascular events, and malignant tumors^5^, although we did not evaluate the effect of Rapa in combination with a CNI on the VEGF network. There is a growing body of evidence that combining mTORi with reduced CNI exposure in de novo kidney transplants has an acceptable side effect profile and is immunologically safe^4,8–10,39^. Particularly, there was no indication to increased rates of DGF associated with early use of mTORi^5,26^.

Taken together, our study does not raise a safety signal that mTOR inhibition with Rapa at doses used in modern immunosuppressive protocols after kidney transplantation negatively interferes with the VEGF network that is crucial for successful recovery from preservation/reperfusion injury. Moreover, we provide a rational to evaluate a possible role for therapeutic enhancement of VEGF signaling peritransplant to further improve outcomes. In the light of recent clinical trials, mTORi may be considered in all transplant recipients with low to moderate immunologic risk.

## Methods

### Animals and transplantation surgery

All surgical and experimental procedures were approved by local authorities (Landesamt für Gesundheit und Soziales, LaGeSo, Berlin, Germany) and were in accordance with the guidelines of the American Physiological Society. The ARRIVE guidelines were met. 10-weeks old inbred male Fischer (F344) and Lewis (Lew, RT1) rats (Harlan-Winkelman, Sulzbach, Germany) weighing 150–200 g were kept at 24 °C with regular lighting conditions (lights on 6:00–18:00) with free access to tap water and standard rat diet (C-1000, Altromin, Lage, Germany). Fischer rats served as kidney donors and were prepared as described previously^17^. After in situ perfusion with 5 mL pre-cooled University of Wisconsin (UW) solution through the cannulated aorta, the explanted kidney was placed in cold UW (4 °C) for 2 h. The Lewis recipients were anesthetized with isoflurane and underwent bilateral nephrectomy followed by orthotopic implantation of the left donor kidney. Anastomoses of the artery, vein and ureter were done end-to-end with 10–0 polypropylene (Prolene, Ethicon, Norderstedt, Germany) sutures within 30 min^18^.

Rapa (LC Laboratories, Woburn, MA, USA) was administered by gavage from a 1 mg/mL stock solution. A loading dose of 3 mg/kg was applied followed by daily maintenance doses of 1.5 mg/kg. Rapa trough levels of 4.30 ± 0.64 ng/mL were achieved.

As previously described^19^, rats were placed in metabolic cages for collection of 24-h urine samples. When animals were killed at the indicated time points venous blood was collected for automated measurements of creatinine and urea in the university’s central laboratory facility and kidney grafts were harvested.

### Histology, immunohistochemistry, and morphometric quantification

Histological and immunohistochemistry techniques followed previously described protocols^18^. We used the alkaline phosphatase/anti-alkaline phosphatase (APAAP) complex method (DakoCytomation, Hamburg, Germany) for immunostaining. Acetone-fixed cryosections (6 µm) were used for analysis with antibodies directed against rat endothelial cell antigen (RECA; Abcam, Cambridge, UK), endothelial NO-synthase (eNOS; Thermo Fisher Scientific, Waltham, MA, USA), and VEGF-A (R&D Systems, Minneapolis, MN, USA) using the neufuchsin-naphtol-As-Bi-phosphate substrate (Merck, Darmstadt, Germany). Proliferating cell nuclear antigen (PCNA; Zymed Laboratories Inc., San Francisco, CA, USA) was detected in paraffin Sects. (4 µm) of formalin fixed tissue with amino ethyl carbazole (AEC) as the chromogen (DakoCytomation). Negative control staining was performed by incubation with corresponding isotype controls instead of primary antibody. Hematoxylin counterstain was applied to all sections after development of the antibody signal.

For all parameters, 10 randomly chosen fields of view (FOV) at 400× magnification were evaluated and summarized to obtain a single mean value for each individual rat. The area stained by RECA and eNOS antibodies was measured using a computer-assisted morphometry unit (axiocam HR with axiovision 4.4 software, Zeiss/Kontron, Göttingen, Germany) and expressed as the percentage of the total area. PCNA positive tubular cells were assessed as percentage of all tubular cells.

### Cell culture

Immortalized rat renal proximal tubular cells (RPTC) were generously provided by Julie Ingelfinger (Pediatric Nephrology Laboratory, Harvard Medical School, Boston, MA, USA). RPTC were cultured in Dulbecco’s Modified Eagle’s Medium (DMEM; Biochrom, Berlin, Germany) supplemented with 10% fetal calf serum, 100 U/mL penicillin, 100 µg/mL streptomycin, and 2 mmol/L glutamine at 37 °C with 5% CO_2_ in a humidified atmosphere. Cells were passaged before reaching confluence.

RPTC were seeded at a density of 250,000 cells per well in 6-well plates for RNA extraction or 10,000 cells per well in 96-well plates for BrdU and MTT assays in complete DMEM and were allowed to adhere overnight. After cells were exposed to serum-free medium for 24 h they were treated with Rapa at the indicated concentrations or with ethanol as a vehicle control for 4, 8, or 24 h at normoxia and hypoxia in parallel.

To create hypoxic conditions, culture dishes were placed in an air-tight hypoxia chamber with constant 95% N_2_/5% CO_2_ in a humidified atmosphere at 37 °C.

Rapa (LC Laboratories) was dissolved in ethanol and stock solutions with concentrations of 2 µmol/L, 12 µmol/L, and 100 µmol/L were prepared.

### Quantitative real-time PCR

Total RNA was extracted from deep frozen graft tissue samples or cultured cells with TRIzol (Invitrogen, Carlsbad, CA, USA) and purified following standard procedures. Isolated RNA was checked for integrity by agarose gel electrophoresis with ethidium bromide staining and spectrometrically quantified. Complementary DNA (cDNA) was obtained from 1 µg of RNA using the PCR Core Kit and random hexamer primers (Applied Biosystems, Foster City, CA, USA) following to the manufacturer's protocol.

The Light Cycler PCR and detection system (Roche, Mannheim, Germany) was used for amplification and online quantification. Specific primer pairs (TIB Molbiol, Berlin, Germany) were designed to detect the following target transcripts: eNOS, forward 5′-TGA CCC TCA CCG ATA CAA CA, reverse 5′-CTG GCC TTC TGC TCA TTT TC; VEGF-A, forward 5′-TGC ACC CAC GAC AGA AGG GGA, reverse 5′-TCA CCG CCT TGG CTT GTC ACA T; VEGF-R1, forward 5′-CAA GGG ACT CTA CAC TTG TC, reverse 5′-CCG AAT AGC GAG CAG ATT TC; VEGF-R2, forward 5′-GCC AAT GAA GGG GAA CTG AAG AC, reverse 5′-TCT GAC TGC TGG TGA TGC TGT C; neuropilin-1, forward 5′-GAT TCC CTG AAG TTG GCC CT, reverse 5′-TCT CCT GGT GTC CAC CCG TT. Glyceraldehyde-3-phosphate dehydrogenase (GAPDH) was used as an internal standard (forward 5′-CCA TCT TCC AGG AGC GAG AT, reverse 5′-GAT GAC CTT GCC CAC AGC CT). The PCR mixture consisted of H_2_O, *Taq* polymerase, 3 mM magnesium chloride, Master Sybr Green Mix® (Roche), and specific primers. cDNA corresponding to 0.1 µg RNA was analyzed per reaction. Melting curve analyses were performed to verify the specificity of the reactions. Run data were analyzed with the quantification program Quant V3·0 using the delta-CT method.

### VEGF-A enzyme immunoassay (EIA)

Cell culture supernatants were cleared by centrifugation for 3 min at 100×*g*. Concentration of VEGF-A was determined with a commercial human VEGF EIA that crossreacts with rat VEGF-A (PromoKine C-64407; PromoCell, Heidelberg, Germany) following the manufacturer′s protocol.

### BrdU incorporation for assessment of cell proliferation

RPTC in 96-well were exposed to the indicated Rapa concentrations or ethanol as vehicle control in serum-free DMEM for 24 h. 5-Bromo-2′-deoxy-uridine (BrdU) was added 1:1,000 for the last 2 h. BrdU incorporation into newly synthetized DNA was measured as a surrogate for proliferation using the BrdU cell proliferation kit (Roche) according to the manufacturer’s instructions. Each measurement consisted of three replicates.

### MTT assay for metabolic activity

RPTC in 96-well plates cells were exposed to the indicated concentrations of Rapa or ethanol as the vehicle control for 24 h. 3-(4,5-Dimethyl-2-thiazolyl)-2,5-diphenyl-2H-tetrazoliumbromid (MTT; Merck) at a concentration of 5 g/L in sterile 0.9% NaCl solution was added to a final concentration of 1.5 mmol/L for the last 60 min. After washing with PBS, formazan crystals formed by metabolically active cells were solubilized in 100 µl isopropanol/4 mol/L HCl. Absorbance was measured in a microplate reader at 570 nm. Background absorbance was subtracted and means of five replicates were calculated.

### Statistical analysis

Quantitative results are expressed as means ± SEM. Numbers of animals or independent replicates are given in each figure legend. Treatment groups and different time points or normoxic versus hypoxic conditions and different rapa concentrations were compared with the two-way analysis of variance (ANOVA). Bonferroni’s multiple comparisons test was used for post-testing. Statistical significance was considered at a two-sided *P* value of < 0.05. GraphPad Prism 5.0 (GraphPad Software, La Jolla, CA, USA) for Windows was used for all analyses.

## Data Availability

The datasets generated and analyzed during the current study are available from the corresponding author on reasonable request.
